# Season of Conception in Rural Gambia Affects DNA Methylation at Putative Human Metastable Epialleles

**DOI:** 10.1371/journal.pgen.1001252

**Published:** 2010-12-23

**Authors:** Robert A. Waterland, Richard Kellermayer, Eleonora Laritsky, Pura Rayco-Solon, R. Alan Harris, Michael Travisano, Wenjuan Zhang, Maria S. Torskaya, Jiexin Zhang, Lanlan Shen, Mark J. Manary, Andrew M. Prentice

**Affiliations:** 1 Department of Pediatrics, Baylor College of Medicine, USDA/ARS Children's Nutrition Research Center, Houston, Texas, United States of America; 2 Department of Molecular and Human Genetics, Baylor College of Medicine, Houston, Texas, United States of America; 3 MRC International Nutrition Group, London School of Hygiene and Tropical Medicine, London, United Kingdom; 4 Department of Ecology, Evolution, and Behavior, University of Minnesota, St. Paul, Minnesota, United States of America; 5 Department of Biostatistics and Applied Biomathematics, The University of Texas M. D. Anderson Cancer Center, Houston, Texas, United States of America; Queensland Institute of Medical Research, Australia

## Abstract

Throughout most of the mammalian genome, genetically regulated developmental programming establishes diverse yet predictable epigenetic states across differentiated cells and tissues. At metastable epialleles (MEs), conversely, epigenotype is established stochastically in the early embryo then maintained in differentiated lineages, resulting in dramatic and systemic interindividual variation in epigenetic regulation. In the mouse, maternal nutrition affects this process, with permanent phenotypic consequences for the offspring. MEs have not previously been identified in humans. Here, using an innovative 2-tissue parallel epigenomic screen, we identified putative MEs in the human genome. In autopsy samples, we showed that DNA methylation at these loci is highly correlated across tissues representing all 3 embryonic germ layer lineages. Monozygotic twin pairs exhibited substantial discordance in DNA methylation at these loci, suggesting that their epigenetic state is established stochastically. We then tested for persistent epigenetic effects of periconceptional nutrition in rural Gambians, who experience dramatic seasonal fluctuations in nutritional status. DNA methylation at MEs was elevated in individuals conceived during the nutritionally challenged rainy season, providing the first evidence of a permanent, systemic effect of periconceptional environment on human epigenotype. At MEs, epigenetic regulation in internal organs and tissues varies among individuals and can be deduced from peripheral blood DNA. MEs should therefore facilitate an improved understanding of the role of interindividual epigenetic variation in human disease.

## Author Summary

There is growing interest in the possibility that interindividual epigenetic variation plays an important role in a broad range of human diseases. The tissue-specificity of epigenetic regulation, however, will in many cases make it difficult to obtain the appropriate tissues in which to perform large-scale studies linking epigenetic dysregulation to disease. We have used an innovative two-tissue DNA methylation screen to identify genomic regions that exhibit interindividual epigenetic variation which occurs systemically—i.e. similarly in all tissues. Such regions—called metastable epialleles—have previously been identified in mice because they cause visible phenotypic variation amongst genetically identical individuals. Indeed, we found that even monozygotic twins show substantial epigenetic discordance at these loci. Further, we show that, as in mice, establishment of DNA methylation at these putative human metastable epialleles is labile to maternal environment around the time of conception. Metastable epialleles should facilitate an improved understanding both of the role of interindividual epigenetic variation in human disease and of the effects of early environment on the establishment of human epigenotype.

## Introduction

Epigenetic mechanisms maintain mitotically heritable differences in gene expression potential without alterations in DNA sequence [1], enabling the diverse cell types of multicellular organisms to stably regulate appropriate patterns of gene expression. The established role of epigenetic mechanisms in cancer and various developmental syndromes has spurred increasing interest in the role of epigenetic dysregulation in a broad range of human diseases including neurological disorders, cardiovascular disease, diabetes, and obesity. A major obstacle to studying epigenetics and human disease, however, is the inherent tissue specificity of epigenetic regulation. In studies of genetic epidemiology, DNA from peripheral blood can be used to assay for a genetic variant present throughout the body. Conversely, epigenetic regulation [2], [3] (and hence dysregulation) may be tissue- and cell-type specific [4], [5]; in many cases, therefore, epigenetic information present in easily obtainable biopsy samples will not provide insights into the epigenetic etiology of disease. Another major obstacle is that interindividual epigenetic variation may often be a consequence of genetic variation [6], making it difficult to disentangle epigenetic and genetic causes of disease.

Hence, genomic loci at which systemic interindividual epigenetic variation occurs independently of genotype would offer major opportunities to advance our understanding of epigenetics and human disease. Such loci have been identified in the mouse; at murine metastable epialleles (MEs) epigenetic regulation is established stochastically in the early embryo then maintained in all germ-layer lineages, resulting in dramatic and systemic interindividual variation in locus-specific epigenetic regulation. Murine MEs cause obvious phenotypic variation among genetically identical mice. For example, the *Agouti viable yellow* (*A^vy^*) ME affects the expression of the *Agouti* gene which regulates fur pigmentation; isogenic mice heterozygous for *A^vy^* range from yellow to mottled to brown [7]. Similarly, the *Axin Fused* (*Axin^Fu^*) ME confers epigenetic stochasticity upon *Axin*, resulting in interindividual variation in tail kinking among isogenic *Axin^Fu^* heterozygous mice [8]. Rather than affecting fur color or tail development, however, MEs in the human genome could affect individual susceptibility to various diseases. Indeed, because agouti protein binds antagonistically to the melanocortin 4 receptor in the hypothalamus [9], yellow *A^vy^/a* mice become hyperphagic and obese, illustrating how epigenetic dysregulation at MEs can result in metabolic disease.

Maternal nutrition and other environmental exposures before and during pregnancy influence the stochastic establishment of epigenetic regulation at murine MEs, with permanent phenotypic consequences [10]–[12]. Hence, if MEs can be identified in humans, they would not only facilitate an advanced understanding of the role of epigenetics in human disease, but also provide excellent candidate loci at which to test epigenetic pathways in the developmental origins hypothesis, which proposes that early environmental influences affect developmental mechanisms, causing permanent metabolic changes that affect risk of adult disease [4], [13]. Of various interacting epigenetic mechanisms including cytosine methylation in DNA, covalent histone modifications, and autoregulatory DNA binding proteins, DNA methylation is recognized as the most stable epigenetic mark [14], making it a prime candidate to mediate the life-long epigenetic changes postulated in the developmental origins paradigm [5], [13].

Here, we have designed an innovative epigenomic screen based upon the epigenetic characteristics of murine MEs, and have screened for MEs in the human genome. We provide evidence that MEs do exist in humans. At the loci we identified, systemic interindividual variation in DNA methylation was confirmed in autopsy samples, and stochastic establishment of epigenotype was supported by epigenetic discordance within monozygotic (MZ) twin pairs. Further, by studying children conceived during different seasons in rural Gambia we show that, as in mice, developmental establishment of DNA methylation at such sites is responsive to maternal environment around the time of conception.

## Results

**Figure 1:**
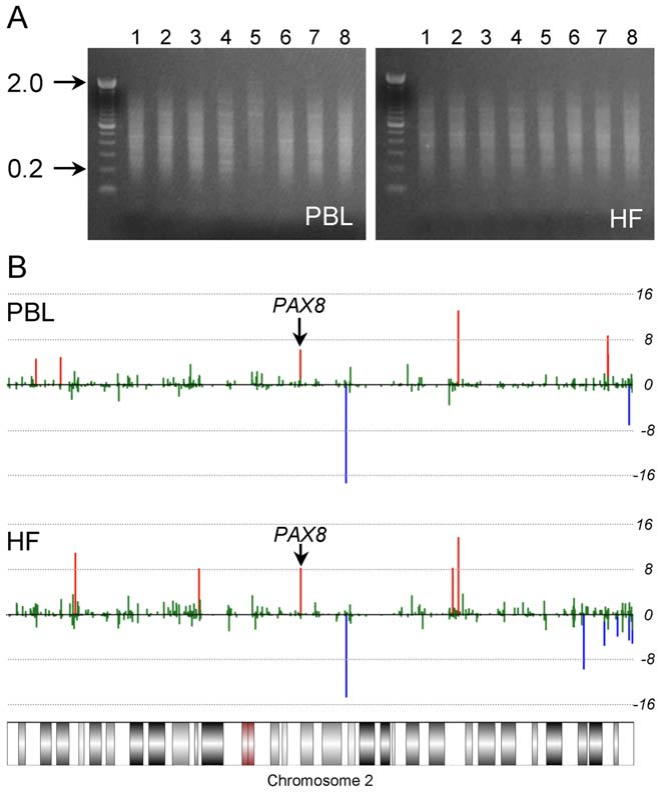
Two-tissue MSAM screen. (*A*) Agarose gel images showing MSA product from PBL and HF genomic DNA of the 8 individuals included in the screen. MSA amplifies methylated *SmaI/XmaI* intervals ranging from <100 bp to 2 kb. (*B*) Results of the MSAM screen at chromosome 2 for one pair of PBL and HF interindividual cohybridizations. Each bar represents the average of all probes within a single *SmaI/XmaI* interval on chromosome 2. The y axis is a log_10_ transformation of the P value of the interindividual signal ratio. Red and blue bars represent positive and negative interindividual differences, respectively, with P<10^−4^. *SmaI/XmaI* intervals showing concordant interindividual differences in PBL and HF are ME candidates; the location of the hit at *PAX8* is indicated.

We devised a human genome-scale screening approach based on a definitive characteristic of murine MEs: systemic interindividual variation in DNA methylation [11], [12]. Genomic DNA from peripheral blood leukocytes (PBL) and hair follicles (HF) (mesodermal and ectodermal lineages, respectively) of 8 healthy Caucasian adults was screened for interindividual differences in DNA methylation by methylation-specific amplification microarray [15] (MSAM). We employed a parallel, 2-tissue interindividual cohybridization design: the same four interindividual comparisons (matched for age and sex) were performed in both PBL and HF DNA (Figure 1). Consistent with previous studies [2], [16], most CpG sites assayed did not show measurable interindividual differences in methylation (Table S1). Moreover, interindividual differences were more often observed in a single tissue than in both tissues (Table S1). Nonetheless, our approach identified 107 genomic loci exhibiting concordant interindividual MSAM differences in both tissues (Table S2A).

MSAM is based upon serial digestion of genomic DNA with the methylation sensitive/insensitive isoschizomers *SmaI* and *XmaI*; our screen could therefore detect genetic variation in addition to systemic epigenetic variation. Indeed, initial attempts to validate several candidates by bisulfite pyrosequencing failed to detect differences in DNA methylation and instead identified single nucleotide polymorphisms (SNPs) within *SmaI/XmaI* sites. We bioinformatically annotated all potentially informative human *SmaI/XmaI* intervals with a known SNP within either *SmaI/XmaI* site (CCCGGG). SNPs that introduce a *SmaI/XmaI* site within a consensus *SmaI/XmaI* interval also could affect the MSAM signal, and were likewise annotated. Of 107 *SmaI/XmaI* intervals originally identified in our screen, 34 were associated with *SmaI/XmaI* SNPs, a significant over-representation (P = 1.3×10^−8^). After excluding these (Table S2B), we observed that the remaining 73 candidate MEs tended to localize in subtelomeric regions (Figure S1A). Given the propensity for copy number variation in subtelomeric regions [17], we identified all potentially informative *SmaI/XmaI* intervals located within known human copy number variants and segmental duplications. Nearly half (35) of the remaining 73 candidate MEs were located within these genetically variable regions, many more than expected by chance (P = 2.1×10^−23^). After excluding these (Table S2C), the subtelomeric localization was eliminated (Figure S1B).

Excluding all candidate *SmaI/XmaI* intervals associated with known SNPs, copy number variants, and segmental duplications is extremely conservative, and likely excludes MSAM hits that are in fact caused by interindividual variation in DNA methylation. Indeed, 2 hits in which interindividual DNA methylation differences had already been validated before we performed the bioinformatic filtering were among the affected intervals: the interval at *ZNF696* is associated with a *SmaI/XmaI* SNP, and that at *FLJ20433* is within a copy number variant. These 2 loci were retained in the final list of candidate MEs, bringing the number to 40. Of 13 we analyzed by bisulfite pyrosequencing, interindividual variation in PBL and HF DNA methylation was confirmed in 8. (Failure to validate could be caused by uncharacterized SNPs and CNVs, inability to assay both of the informative *SmaI/XmaI* sites, or low overall methylation levels.)

**Figure 2:**
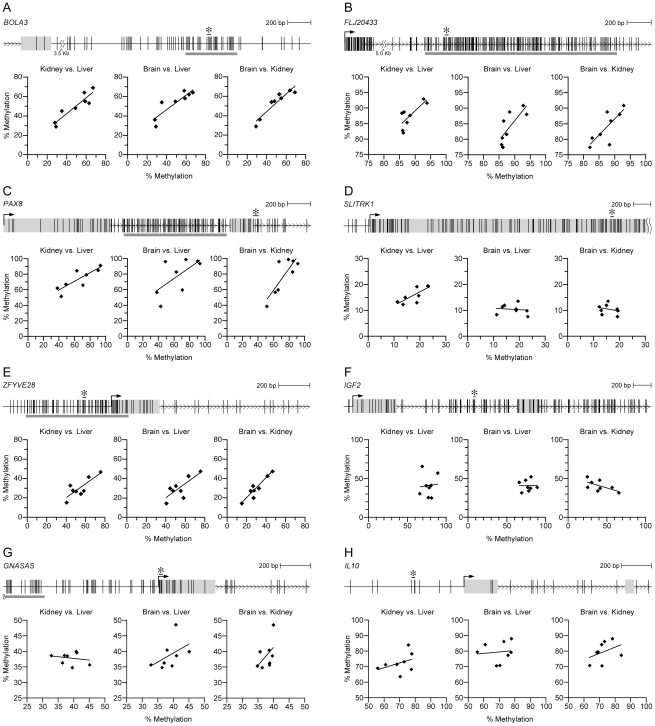
Candidate MEs, but not control genes, show systemic interindividual variation in DNA methylation. (A–E) Scatter plots illustrating inter-tissue correlation of interindividual differences in DNA methylation at candidate MEs *BOLA3*, *FLJ20433*, *PAX8*, *SLITRK1*, and *ZFYVE28*. The top of each panel indicates the genomic region. Vertical lines represent CpG sites, and gray horizontal bars represent CpG islands. The CpG sites covered by each pyrosequencing assay are indicated by an asterisk. All 5 candidate MEs show significant inter-tissue correlation, indicating systemic interindividual variation in DNA methylation. (F–H) Control genes *IGF2*, *GNASAS*, and *IL10* exhibit interindividual variation in DNA methylation comparable to that of the candidate MEs, but there is no significant inter-tissue correlation. (Correlation coefficients and P values for all regions are provided in Table S3.)

Our screen was performed using DNA from Caucasians, using 2 tissues that can be sampled relatively non-invasively. To verify concordance across tissues derived from all 3 germ layers, and determine if interindividual epigenetic variation at candidate MEs is conserved across genetically divergent populations, post-mortem liver, kidney and brain tissue was obtained from 8 Vietnamese motor vehicle accident victims (healthy donors). Of the 8 genomic regions with confirmed interindividual variation in DNA methylation in the Caucasian PBL and HF samples, 5 (*BOLA3*, *FLJ20433*, *PAX8*, *SLITRK1*, and *ZFYVE28*) showed interindividual variation that was highly correlated among liver, kidney, and brain in the Asian sample (Figure 2A–2E, and Table S3). (*SLITRK1* was exceptional in that methylation in brain did not correlate with that in liver and kidney (Figure 2E). This is potentially analogous to the murine *Axin^Fu^* ME, at which DNA methylation in tail differs from that in all other tissues [11].) For comparison, we similarly analyzed regions within *IGF2*, *GNASAS*, and *IL10*, at which DNA methylation in PBL DNA has been associated with early famine exposure [18], [19]. Although substantial interindividual variation in DNA methylation was confirmed at these loci, not a single statistically significant inter-tissue correlation was found (Figure 2F–2H, and Table S3).

To identify specific genomic characteristics that may confer the special epigenetic behavior of these loci, we bioinformatically compared 6 kb windows encompassing the 40 putative ME *SmaI/XmaI* intervals and 5000 ‘control’ intervals on the array. We assessed several characteristics of associated CpG islands, as well as the distribution of various classes of tranposable elements (Figures S2, S3, S4, S5, S6, S7, S8, S9). The only significant finding was in the distribution of long-terminal repeat (LTR) retrotransposons; these were depleted at and distributed symmetrically around control intervals, but preferentially localized downstream of putative ME intervals (P = 0.001) (Figure S7). Although clearly insufficient to explain epigenetic metastability, this finding is noteworthy in that nearly all known murine MEs are associated with intracisternal A particle LTR-retrotransposons [20], [21].

Our aim was to identify interindividual epigenetic variation that occurs stochastically; the multiple-tissue screening approach could, however, also detect epigenetic variation associated with genetic variation [6], [22], [23]. Indeed, while performing pyrosequencing validation of one candidate ME, *ZNF696*, a proximal SNP was identified that explained most of the interindividual variation in methylation (Figure S10). To attempt to rule out such effects, one could map the genomic region flanking each candidate ME to identify haplotype blocks correlated with methylation status. But effects of genetic variation on DNA methylation can occur in cis over tens or even hundreds of kb [23], [24], or in trans [25]. By genetic mapping alone, therefore, it is virtually impossible to exclude that the systemic interindividual epigenetic variation at these select loci is attributable to genetic variation.

Epigenetic discordance within pairs of MZ twins would provide support that interindividual epigenetic variation at our candidate MEs is truly stochastic. We measured DNA methylation at *BOLA3*, *FLJ20433*, and *PAX8* in buccal DNA from 23 pairs of MZ twins (Figure S11). At *PAX8*, although there was significant inter-twin correlation, about half of the variance in DNA methylation was not shared by co-twins. At *BOLA3* and *FLJ20433* there was no inter-twin correlation. These data provide evidence that the interindividual epigenetic variation at our candidate MEs is not genetically mediated.

Another way to determine whether the epigenetic variation at these loci is truly stochastic is to test for an early environmental effect. Unlike interindividual epigenetic variation that is secondary to genetic variation, the stochastic epigenetic variation at *bona fide* MEs can be influenced by maternal nutrition during early embryonic development [10]–[12]. Demonstrating an effect of periconceptional nutrition on DNA methylation at the identified genomic regions would therefore provide further support that they are MEs. The rural villagers in West Kiang, the Gambia are subsistence farmers whose nutritional status varies dramatically by season. During the rainy season (July–November) depletion of food stores from the previous harvest, combined with an intense agricultural workload, causes negative energy balance and consequent effects on reproductive outcomes [26]. Relative to the dry season, average birth weight during the rainy season is 200–300 g lower and the incidence of small for gestational age infants is doubled [27]. Importantly, seasonal effects on fetal development persist to affect adult mortality in this population [28], but the underlying biologic mechanisms remain unknown.

To test the hypothesis that periconceptional nutrition affects developmental establishment of DNA methylation at candidate MEs, we compared DNA methylation in peripheral blood leukocytes (PBL) of Gambian children conceived during either the dry or the rainy season. Effects of seasonality vary from year to year; we therefore used retrospective birth weight data to identify 1991, 1994, 1995, 1997, and 1998 as years with strong effects of seasonality (Figure S12). Individuals conceived during August–September (rainy season) were compared with those conceived during March–May (dry season), matching for sex and year of conception (n = 30/season). Blood was collected from the children at age 8.9±0.5 years (mean ± sem); age at blood collection did not differ between the season of conception groups. Preliminary analyses of the DNA methylation data showed a highly significant (season of conception) × (year of conception) interaction (P = 0.005), indicating that the effect of seasonality was not consistent in all years. Examining the effects in each year indicated that 1997 was an outlier. Excluding individuals conceived in 1997 eliminated the (season of conception) × (year of conception) interaction (P = 0.17) and left n = 25 individuals per season, representing four years (1991, 1994, 1995, and 1998) in subsequent analyses.

**Figure 3:**
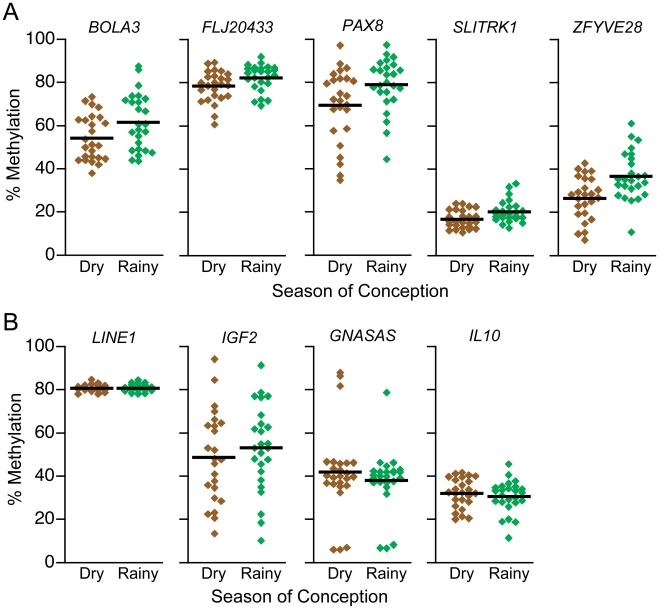
DNA methylation at putative MEs is influenced by season of conception in the Gambia. (A) Percent methylation at putative MEs *BOLA3*, *FLJ20433*, *PAX8*, *SLITRK1*, and *ZFYVE28* in PBL DNA of Gambian children, relative to season of conception. Each circle represents one individual, and the black lines represent group means (n = 25/group). At all 5 genomic regions, DNA methylation is higher in individuals conceived in the nutritionally challenged rainy season (*BOLA3* P = 0.03, *FLJ20433* P = 0.03, *PAX8* P = 0.02, *SLITRK1* P = 0.006, *ZFYVE28* P = 0.002; overall P = 0.0001). (B) At generic LINE1 elements, as well as at 3 control genes (*IGF2*, *GNASAS*, and *IL10*), DNA methylation in these same individuals is not correlated with season of conception (overall P = 0.24), indicating that establishment of epigenotype at the regions we have identified is particularly labile to periconceptional environment.

Since maternal supplementation with dietary methyl donors increases DNA methylation at MEs in murine offspring [10]–[12], we anticipated that DNA methylation would be reduced in individuals conceived during the nutritionally challenged rainy season. We found the opposite. At all 5 putative MEs, DNA methylation was significantly higher among individuals conceived during the rainy season (Figure 3A). The overall effect of season of conception on DNA methylation at the 5 MEs combined was highly significant (P = 0.0001). (Detailed statistical analyses provided in Text S1.) Unlike persistent changes in DNA methylation associated with periconceptional famine exposure [18], [19] the effect sizes at the genomic regions we identified were not subtle; rainy season conception was associated with absolute methylation increments of over 10% at both *PAX8* and *ZFYVE28* (Figure 3A). To determine if the association of season of conception with DNA methylation might be due to chance genetic differences between the groups (such as, for example, differences in one carbon metabolism), we compared DNA methylation at generic LINE1 elements (an indicator of genome-wide methylation [29]) and the same 3 ‘control’ genes studied in the Asian sample (*IGF2*, *GNASAS*, and *IL10*). Contrary to large studies which have associated early famine exposure with subtle persistent changes in DNA methylation at *IGF2*, *GNASAS*, and *IL10* [18], [19], we found no effect of season of conception in the non-ME control regions, either singly or combined (Figure 3B), indicating that developmental establishment of DNA methylation at MEs is exceptionally sensitive to maternal environment.

The overall effect of season of conception at these putative MEs is especially compelling given that each individual's DNA methylation at one was generally not predictive of methylation at others (Table S4), meaning that stochasticity at these genomic regions is not coordinated. Underscoring the broad relevance of these findings, the genomic loci we identified exhibit similar epigenetic behavior across genetically distinct human populations (Figure S13) indicating that they are ancestral features of the human genome.

## Discussion

Murine MEs have attracted extensive study because of their mysterious ability to cause dramatic phenotypic variation among isogenic animals [7], [21], [30], [31]. Viewed by some as an epigenetic oddity, however, their relevance to humans has been questioned [32]. Here, we have for the first time identified elements that are likely to be human MEs, which are characterized by stochastic and systemic interindividual epigenetic variation. These loci exhibit similar interindividual variation in DNA methylation across tissues derived from all 3 germ layers of the early embryo, indicating setting of epigenotype prior to gastrulation. Epigenetic discordance at these genomic loci within MZ twin pairs indicates that establishment of their epigenetic state is determined not genetically, but stochastically. Further, as at murine MEs [10]–[12], developmental establishment of epigenotype at these loci is exquisitely sensitive to maternal periconceptional environment.

Interindividual epigenetic variation that is both systemic and stochastic has not been previously documented in humans. In many cases human interindividual epigenetic variation has been found to be caused by genetic variation [6], [22], [23]. Recent studies of MZ twin pairs have identified epigenetic differences that occur independent of genetic variation [33], [34], but since those differences were studied only in specific tissues it is not clear if they occur systemically. Our results suggest that interindividual epigenetic variation is more often tissue-specific than systemic. Only about half of the *SmaI/XmaI* intervals showing interindividual variation in PBL, and 15% of those showing interindividual variation in HF, exhibited consistent interindividual variation in both tissues (Table S1).

A key issue is whether establishment of epigenotype at the loci we have identified is truly stochastic. One might argue that the systemic interindividual differences in DNA methylation could be caused by genetic variation, but two pieces of evidence suggest otherwise: the epigenetic discordance within MZ twin pairs, and the effect of season of conception. We must, however, note some caveats. Since we studied DNA methylation in only one tissue from MZ twins, we can not definitively say the observed MZ twin discordance arose in the very early embryo. Future studies should examine ME methylation in MZ twins using DNA from multiple tissues representing the three embryonic germ layers. Likewise, in the Gambian studies, we studied DNA methylation in only one tissue. Hence, although the most parsimonious interpretation of the season of conception effect on PBL DNA methylation is an environmental influence on the early embryo, other interpretations are plausible. For example, if 3 months of age (i.e. 1 year after conception) is a critical window for developmental epigenetics in PBL, there could be a seasonal influence on these processes. Alternatively one could postulate reverse causality, whereby physiological changes induced by seasonal influences on development lead to secondary alterations in DNA methylation. Studying the effect of season of conception on ME DNA methylation in multiple tissues (which is currently underway) will test both of these alternative hypotheses. It is unlikely that postnatal seasonal effects or secondary effects of altered physiology would induce similar epigenetic changes in diverse tissues.

Although DNA methylation at the *PAX8* ME was significantly correlated within MZ twin pairs (Figure S11), this does not necessarily indicate a genetic effect on epigenotype. If setting of epigenotype at MEs occurs prior to blastocyst cleavage during MZ twinning, both members of an MZ twin pair could carry concordant epigenetic states at MEs, despite stochastic establishment. Given the different timing of blastocyst cleavage in dichorionic vs. monochorionic MZ twins, examining ME DNA methylation among these different subtypes of MZ twin pairs may prove informative.

The identification of human MEs should advance the study of epigenetics and human disease. Because individually-variable DNA methylation at these loci exhibits little tissue-specificity, epigenetic dysregulation in pathophysiologically relevant tissues such as thyroid and brain, for example, can be inferred from PBL DNA. Indeed, among the putative MEs we identified are genes implicated in hypothyroidism (*PAX8*) [35], and Tourette's syndrome (*SLITRK1*) [36]. Such sites therefore represent excellent candidate loci for future studies of epigenetic epidemiology which will utilize existing DNA sample collections to explore associations between epigenetic variation and human disease. Moreover, given their epigenetic lability to early environmental influences, human MEs may enable the elaboration of mechanistic pathways linking early environment to later risk of disease [4], [37]. To the extent that epigenetic variation at MEs is associated with diseases such as cardiovascular disease, type-2 diabetes, and obesity, we may better understand how early nutrition and other environmental exposures predict adult risk of these diseases [5].

By no means should it be inferred that MEs are the sole genomic substrate for early environmental influences on epigenetic regulation. Extensive data from animal [38], [39] and human studies [18], [19] indicate that environmental factors affect epigenetic processes over a broad range of developmental periods, with long-term consequences. Stochastic establishment of epigenotype at MEs, however, does appear to be particularly sensitive to periconceptional environment. For example, by studying 60 famine-exposed humans and their unexposed same sex-siblings, Heijmans et al detected persistent effects of periconceptional famine exposure on PBL DNA methylation at the *IGF2* DMR [18], *GNASAS*, and *IL10* [19]. Here, we show that seasonal variation in periconceptional nutrition – likely a milder perturbation – induced significant changes in DNA methylation at all 5 putative MEs studied, but not at the *IGF2* DMR, *GNASAS*, or *IL10*. Moreover, unlike epigenetic changes that occur only in specific tissues, environmentally-induced epigenetic changes at MEs affect the entire body, and are therefore more likely relevant to human physiology and disease.

Enormous interest in transgenerational epigenetic inheritance has recently been stimulated by provocative data indicating that environmental influences during development might affect the health of subsequent generations [40], [41]. Since transgenerational epigenetic inheritance is known to occur at murine MEs [7], [30], it is logical to consider whether MEs may likewise provide opportunities to understand non-genetic inheritance in humans.

Our findings raise additional questions for future study. First, what causes epigenetic metastability? The stochastic establishment of epigenotype at MEs must fundamentally be a consequence of the genetic sequence in these genomic regions. Indeed, known murine MEs result from transposition of retrotransposons in or nearby genes [21]. But among the genomic loci identified here, no obvious genetic signature of epigenetic metastability was detected. Since our screen was limited to genomic regions containing multiple *SmaI/XmaI* sites, we detected only a subset of human MEs. Our parallel, 2-tissue screening approach is, however, adaptable to various epigenomic platforms and should enable the identification of many more human MEs. It may then be possible to gain a better understanding of the molecular basis of epigenetic metastability. Second, we still know very little about exactly how maternal nutrition before and during pregnancy affects establishment of epigenotype at MEs. Contrary to our expectations, Gambian individuals conceived in the nutritionally challenged rainy season gained DNA methylation at these putative MEs, emphasizing that our original conjecture that hunger would be associated with a functionally-limiting methyl donor deficiency was overly simplistic. In light of earlier findings that maternal blood folate levels paradoxically increase during the rainy season in the Gambia [42] (potentially due to increased consumption of leafy vegetables), our data suggest that rather than energy intake, availability of one-carbon donors is of key importance. Studies in mouse models and humans are currently underway to improve our understanding of how maternal dietary and other environmental exposures (e.g. insecticides [43] or naturally occurring toxins [44]) affect developmental epigenetics in the preimplantation embryo.

In summary, we have provided strong evidence that stochastic establishment of epigenetic regulation occurs at specific human genomic loci, resulting in interindividual epigenetic variation that affects tissues from all 3 germ layers and persists to adulthood. We have shown that seasonally variable maternal periconceptional exposures affect this stochastic process. Systemic and persistent epigenetic imprints at these loci are likely to be found among diverse human populations that experience seasonal variation in nutritional sufficiency [27], [45], [46] or other environmental exposures during early embryonic development.

## Materials and Methods

### Sample collection and DNA isolation

#### Caucasians

Tissue samples from 8 healthy adults (Table S5) were collected in accordance with institutional IRB regulations. Peripheral blood leukocytes were isolated by ficoll gradient centrifugation. Hair follicles (30–50) were obtained by plucking scalp, eyebrow, or shin hair from the same 8 individuals. Tissues were stored at −80°C until isolation of genomic DNA by proteinase-k digestion and phenol-chloroform extraction [47].

#### Asians

Post-mortem liver, kidney, and brain tissues from 8 Vietnamese motor vehicle accident victims (Table S6) were obtained from a human tissue bank (ILSbio, LLC, Chestertown, MD, USA). The tissues were collected under IRB approved protocols ensuring donor confidentiality. Tissues were flash-frozen upon excision and stored at −80°C until isolation of genomic DNA by proteinase-k digestion and phenol-chloroform extraction [47]. Before DNA isolation, tissue was blotted on absorbent paper to remove excess blood.

#### MZ twins

Malawian twins were being followed in a special clinic as part of a larger study of the gut microbiota in malnutrition. Permission for sample collection and testing was obtained from the College of Medicine Research and Ethics Committee, University of Malawi. Saliva samples (Table S7) were collected using foam swabs inserted into the buccal cavity until saturated, usually for 3–5 minutes, and then placed in the Oragene preservative (DNA Genotek Inc, Kanata, Ontario). DNA was isolated as recommended by the manufacturer (DNA Genotek).

#### Gambians

The DNA samples were part of a DNA collection from all the residents of 3 rural villages in West Kiang, namely Keneba, Kantong Kunda and Manduar, the Gambia; the field work and DNA collection have been described [48]. Peripheral blood (5–10 ml) was extracted from consenting subjects (Table S8) according to guidelines established by the Gambia Government/MRC Laboratories Joint Ethics Committee. Children were generally healthy at the time of blood sampling, with no overt clinical infection. DNA extraction was performed in MRC Keneba, by a salting-out procedure [49]. DNA samples were transported to the MRC Human Genetics Laboratory at Fajara for quantification and stored at −20°C.

### MSAM screen

MSAM was performed as previously described [15], using a starting quantity of 0.5 µg genomic DNA. MSA products from 2 individuals were differentially dye-labeled and cohybridized to a custom 4×44k array. Array probes were within potentially informative *SmaI/XmaI* intervals (60–1500 bp) and were selected from Agilent's proximal promoter and CpG island probe libraries (Agilent Technologies, Santa Clara, CA, USA). The 43,222 probes on the array cover 19,187 *SmaI/XmaI* intervals (average 2.3 probes/interval). Genomic coordinates are based upon hg18 (NCBI Build 36.1). Relevant details of the microarray experiment, including experimental design, microarray probe listing, and hybridization data sets are available in the GEO database (http://www.ncbi.nlm.nih.gov/geo/) (accession # GSE19823).

Four 2-individual MSAM comparisons (Table S5) were performed using PBL DNA, and the same four 2-individual comparisons were performed using HF DNA (incorporating a dye swap) (Table 1).

**Table 1:** Four 2-individual comparisons performed using HF DNA (incorporating a dye swap).

Comparison	Individuals
A	A_1_ vs. A_2_
B	B_1_ vs. B_2_
C	C_1_ vs. C_2_
D	D_1_ vs. D_2_

The analysis was performed at the level of *SmaI/XmaI* interval; average and median signal intensity, signal ratio, and P value of all probes within each *SmaI/XmaI* interval were calculated. Candidate MEs were identified as follows. For a given paired comparison (say comparison A) we selected all *SmaI/XmaI* intervals with both an average A_1_/A_2_ signal ratio >1.8 or <0.556 and median P<0.0002 in both PBL and HF. All candidates identified in this manner were further filtered to eliminate those in which the 2 tissues showed discordant interindividual ratios in any of the *other* pairwise comparisons; only *SmaI/XmaI* intervals for which the ratio of the PBL:HF signal ratios was >0.445 and <2.25 for all comparisons (A, B, C, D) were retained. (If there is no tissue-specificity in DNA methylation, this ‘ratio of ratios’ equals 1. The maximum departure from this we allowed (2.25) corresponds to a signal ratio of 1.5 in one tissue and 0.667 in the other.) This procedure resulted in the 107 candidate MEs listed in Table S2A.

### Bioinformatic analyses

#### Identification of *SmaI/XmaI* intervals potentially affected by genetic variation

We identified 90,807 *SmaI/XmaI* human genomic intervals between 60–1500 bp based on the hg18 genome version. We then used the UCSC Genome Browser SNPs (129) track (http://genome.ucsc.edu/cgi-bin/hgTrackUi?g=snp129) to identify those in which a SNP disrupts a *SmaI/XmaI* site (N = 10,651), introduces a new *XmaI/SmaI* site within the consensus interval (N = 867), or both (N = 425). We used the Centre for Applied Genomics Database of Genomic Variants, version variation.hg18.v7.mar.2009.txt (http://projects.tcag.ca/variation/) to identify *SmaI/XmaI* intervals located within CNVs, and the UCSC Genome Browser Segmental Duplications track (http://genome.ucsc.edu/cgi-bin/hgTrackUi?g=genomicSuperDups) to identify *SmaI/XmaI* intervals located within segmental duplications. A total of 8631 *SmaI/XmaI* intervals were found to be located within CNVs or segmental duplications.

#### Genomic features in vicinity of MEs

The genomic contexts of the ME intervals (n = 40) and control intervals (n = 5026) were investigated by determining the distance from the midpoint of the intervals to genomic annotations within 3000 bp upstream and downstream. Genomic annotations were obtained from the CpG island and RepeatMasker tracks from the UCSC Genome Browser Human hg18 build (http://genome.ucsc.edu/). All CpG islands in addition to SINE, Alu, LINE, LTR, Simple Repeats and Low Complexity repeats were examined.

### Quantitative analysis of DNA methylation

Site-specific analysis of CpG methylation was performed by bisulfite pyrosequencing. Genomic DNA (0.5–2 µg) was bisulfite modified [38] and pyrosequencing was performed as previously described [50]. The quantitative performance of each pyrosequencing assay was verified by measuring methylation standards comprised of known proportions of unmethylated (whole genome-amplified) and fully methylated (*SssI*-treated) genomic DNA [50]. For initial validation of interindividual variation at candidate *SmaI/XmaI* intervals, DNA methylation was, whenever possible, measured at both *SmaI/XmaI* sites. Subsequent characterization (measurements in other populations, etc) was performed in the vicinity of the *SmaI/XmaI* site showing the greatest interindividual variation.

We assessed interindividual variation in the Caucasian samples at 13 of the 40 candidate MEs identified in the MSAM screen: *AK098581*, *AXIN2*, *BOLA3*, *FLJ20433*, *ITPKB*, *MN1*, *PAX8*, *RCC1*, *SLITRK1*, *SOX10*, *ZNF561*, *ZNF696*, and *ZFYVE28* (primers listed in Table S9). In 5 of these (*AXIN2*, *ITPKB*, *MN1*, *RCC1*, and *SOX10*) the pyrosequencing assays failed to confirm interindividual variation in DNA methylation. *ZNF696* was excluded because it exhibited interindividual variation in methylation that was mostly explained by genetic variation at a neighboring SNP (Figure S10). At the remaining 7 loci we examined tissue-specificity of interindividual variation in the Asian liver, kidney, and brain samples. Five (*BOLA3*, *FLJ20433*, *PAX8*, *SLITRK1*, and *ZFYVE28*) exhibited significant inter-tissue correlations in DNA methylation consistent with MEs. Two that did not (*AK098581* and *ZNF561*) were excluded from further consideration.

### Genotyping

We selected 48 autosomal SNPs with previously demonstrated high reliability for genotyping on the Illumina platform and high minor allele frequency (MAF<0.3) in the Yoruban HapMap population (as the best surrogate we had for the Malawi population). SNPs were selected to be physically distant from each other. These were genotyped on all of the Malawian twin samples. PREST (Pedigree RElationship Statistical Test) was used to estimate the probability of the putative twins sharing 0, 1, or 2 alleles IBD (p0, p1, p2) based on the pairwise analysis of the 48 SNP markers, and the kinship coefficient estimated as phi = 0.25*p1+0.5*p2. In the absence of genotyping error, true MZ twins are expected to have p0 = p1 = 0 and phi = 0.5.

### Statistics

Relative enrichment of candidate ME *SmaI/XmaI* intervals associated with *SmaI/XmaI* SNPs, CNVs, and segmental duplications was analyzed by chi-square tests. Analysis of CGIs and repetitive elements in the vicinity of MEs and control intervals was performed by analysis of variance (ANOVA) (Proc GLM, SAS Version 9.2). Inter-tissue correlations in interindividual variation in methylation were assessed by Pearson correlation analysis (Proc CORR, SAS).

A REML multifactorial ANOVA (JMP Version 8.0) was used to assess factors affecting average methylation in the Gambian season of conception analysis. Methylation was measured multiple times within each individual for each locus and averaged, for a total of 539 averaged observations. Individual and locus were assessed as random factors, with locus nested within locus type (ME or control). Methylation was arcsine transformed to improve normality. Normality was assessed by Shapiro-Wilk Tests for each sample combination of gene and season of conception (18 combinations consisting of 30 individuals each). All samples were statistically indistinguishable from normal distributions, after sequential Bonferroni correction for carrying out 18 simultaneous tests. One interaction, (season of conception) × (locus type) was investigated as an *a priori* test.

## Supporting Information

Figure S1 Localization of ME candidates to sub-telomeric regions is due to genetic variation. ME candidates are indicated by red tick marks. The sub-telomeric localization of SNP-filtered ME candidates (A) is eliminated upon exclusion of known CNVs and segmental duplications (B).
(1.09 MB TIF)

Figure S2 Length of associated CGIs is not different between control (left panel) and ME (right panel) intervals.
(0.41 MB TIF)

Figure S3 Average distance from associated CGIs is not different between control (left panel) and ME (right panel) intervals.
(0.39 MB TIF)

Figure S4 Percent GC of associated CGIs is not different between control (left panel) and ME (right panel) intervals.
(0.45 MB TIF)

Figure S5 Distribution of associated LINE elements is not different between control (left panel) and ME (right panel) intervals.
(0.58 MB TIF)

Figure S6 Distribution of associated SINE elements is not different between control (left panel) and ME (right panel) intervals.
(0.62 MB TIF)

Figure S7 Distribution of associated LTR retrotransposons in the vicinity of control (left panel) and ME (right panel) intervals. Compared to the symmetrical distribution of those near control intervals, LTR retrotransposons occur preferentially downstream of ME intervals (P = 0.001).
(0.51 MB TIF)

Figure S8 Distribution of associated low complexity repeats is not different between control (left panel) and ME (right panel) intervals.
(0.49 MB TIF)

Figure S9 Distribution of associated simple repeats is not different between control (left panel) and ME (right panel) intervals.
(0.51 MB TIF)

Figure S10 Interindividual variation in DNA methylation is predicted by genotype at ZNF696. The top panel shows average percent methylation in Gambian PBL DNA at three CpG sites measured at ZNF696 versus genotype at a neighboring A/G polymorphism (dbSNP build 130 rs28529670) (A/A, n = 25; A/G, n = 10; G/G, n = 5). The box plots indicate median (thick bar), 25th–75th percentiles (box), and 5th–95th percentiles (whiskers). The bottom panel shows representative bisulfite pyrograms for the three genotypes. A reverse sequencing primer was used; the A/G SNP is therefore detected as T/C (upward arrows). The shaded areas of the pyrograms encompass a C within a CpG site. Most interindividual variation in DNA methylation at the locus is explained by genetic variation at the A/G polymorphism.
(0.52 MB TIF)

Figure S11 Correlation within MZ twin pairs for percent methylation at three MEs. Correlation within MZ twin pairs (blue triangles) is compared with correlation among independent replicate PCR and pyrosequencing measurements (red diamonds). Significant inter-twin correlation is found at PAX8 (C), but in every case MZ twins show biological variation that is much greater than the measurement error.
(0.31 MB TIF)

Figure S12 Annual variation in the effect of seasonality on birth weight in Keneba, the Gambia. Average birth weight during the peak rainy season (August-September) is compared with that during the peak dry season (March–May). Whereas some years (such as 1993) show minimal effects of season of birth, we focused on 1991, 1994, 1995, 1997, and 1998 as years with dramatic effects of seasonality.
(0.20 MB TIF)

Figure S13 MEs exhibit similar variation in % methylation across diverse human populations. Average % methylation at BOLA3 (A), FLJ20433 (B), PAX8 (C), SLITRK1 (D), and ZFYVE28 (E) is compared across Asians (n = 8), Caucasians (n = 8), and Gambians (n = 20). The box plots indicate median (thick bar), 25th–75th percentiles (box), and 5th–95th percentiles (whiskers). Despite their genetic dissimilarity, these populations exhibit a similar range of interindividual variation in DNA methylation at each ME.
(0.71 MB TIF)

Table S1 Numbers of SmaI/XmaI intervals that showed tissue-specific, non tissue-specific, or no interindividual variation in MSAM signal.
(0.02 MB XLS)

Table S2 S2A: Candidate MEs, unfiltered. S2B: Candidate MEs, filtered for SmaI/XmaI SNPs. S2C: Candidate MEs, filtered for SmaI/XmaI SNPs and genomic variants.
(0.05 MB XLS)

Table S3 Inter-tissue correlation of interindividual variation in DNA methylation at MEs and control genes.
(0.02 MB XLS)

Table S4 Correlation matrix of average methylation in the Gambian individuals (N = 50) at the five studied MEs. Each box indicates the Pearson correlation coefficient (top) and the P value (bottom). The two significant correlations are highlighted.
(0.02 MB XLS)

Table S5 Caucasian individuals represented in the original MSAM screen.
(0.02 MB XLS)

Table S6 Asian individuals represented in the liver, kidney, brain comparisons.
(0.02 MB XLS)

Table S7 Malawian individuals represented in the MZ twin studies.
(0.03 MB XLS)

Table S8 Gambian individuals represented in the season of conception comparisons.
(0.03 MB XLS)

Table S9 Primers for bisulfite-sequencing assays.
(0.03 MB XLS)

Text S1 Detailed statistical analyses.
(0.48 MB DOC)
