# Supplementary figures and images for: Season of Conception in Rural Gambia Affects DNA Methylation at Putative Human Metastable Epialleles
Source: PLoS Genet. 2010 Dec 23;6(12):e1001252. doi: 10.1371/journal.pgen.1001252 (PMC3009670; doi:10.1371/journal.pgen.1001252)

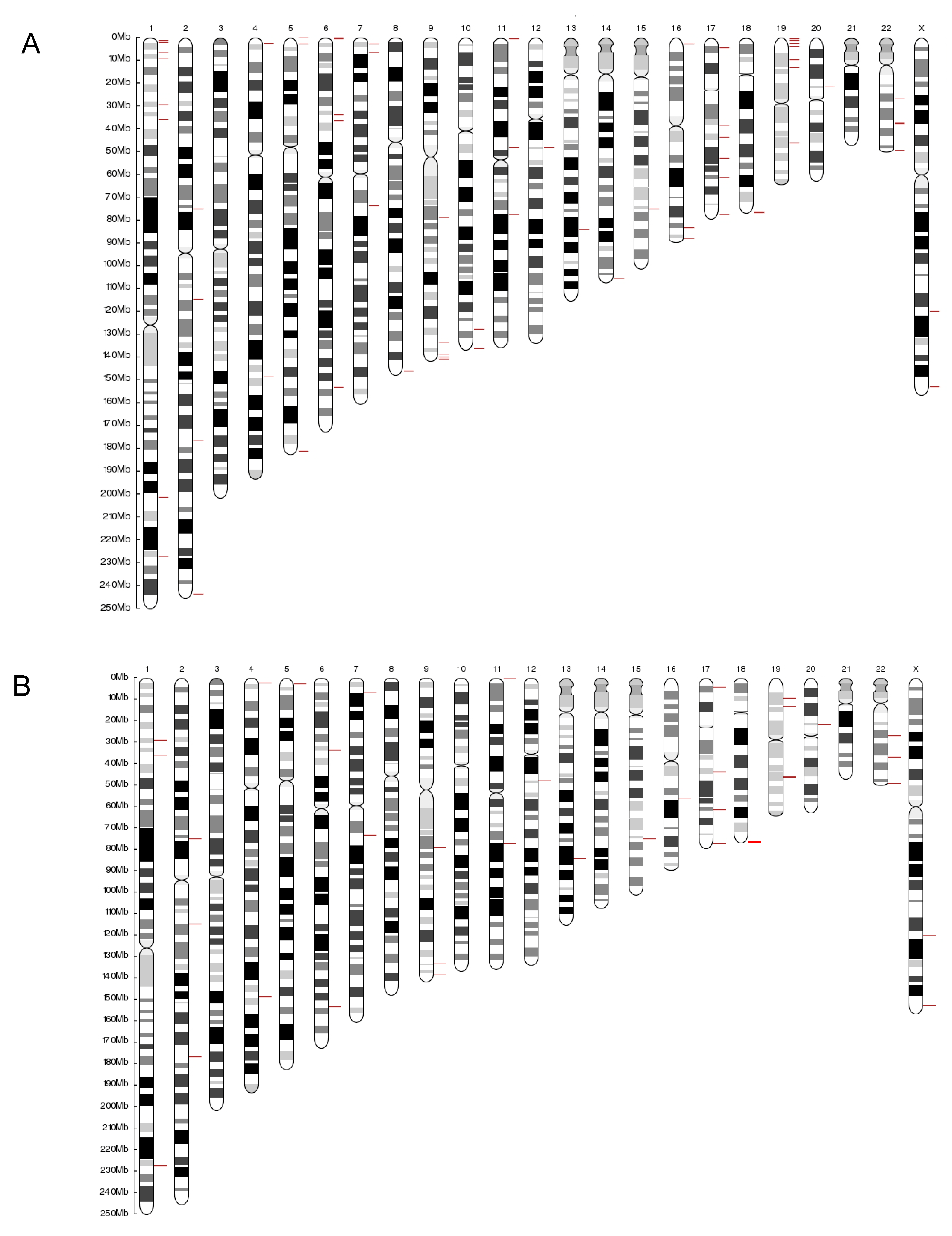

Supplement: Figure S1 — Localization of ME candidates to sub-telomeric regions is due to genetic variation. ME candidates are indicated by red tick marks. The sub-telomeric localization of SNP-filtered ME candidates (A) is eliminated upon exclusion of known CNVs and segmental duplications (B). (1.09 MB TIF) [file pgen.1001252.s001.tif]

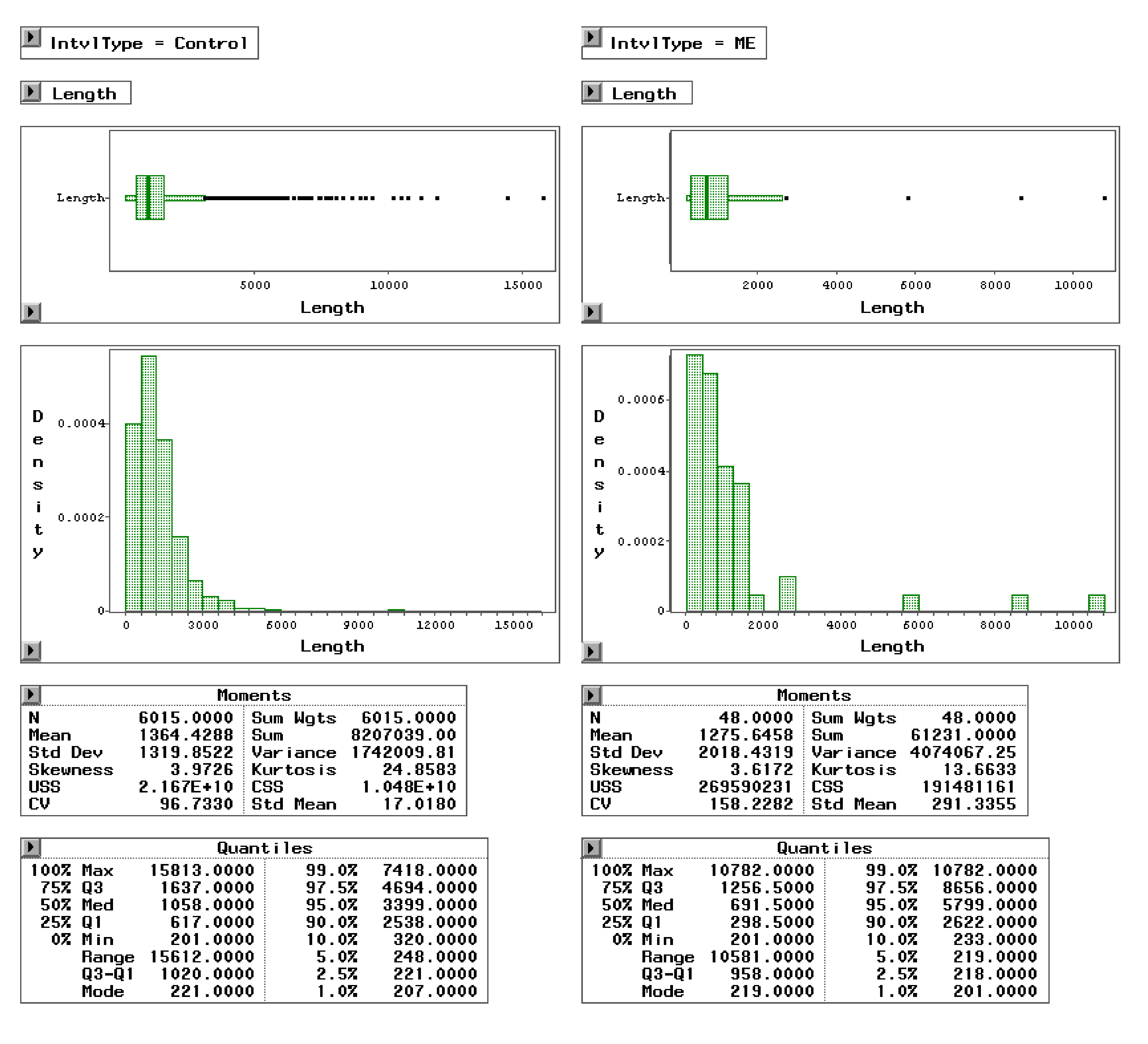

Supplement: Figure S2 — Length of associated CGIs is not different between control (left panel) and ME (right panel) intervals. (0.41 MB TIF) [file pgen.1001252.s002.tif]

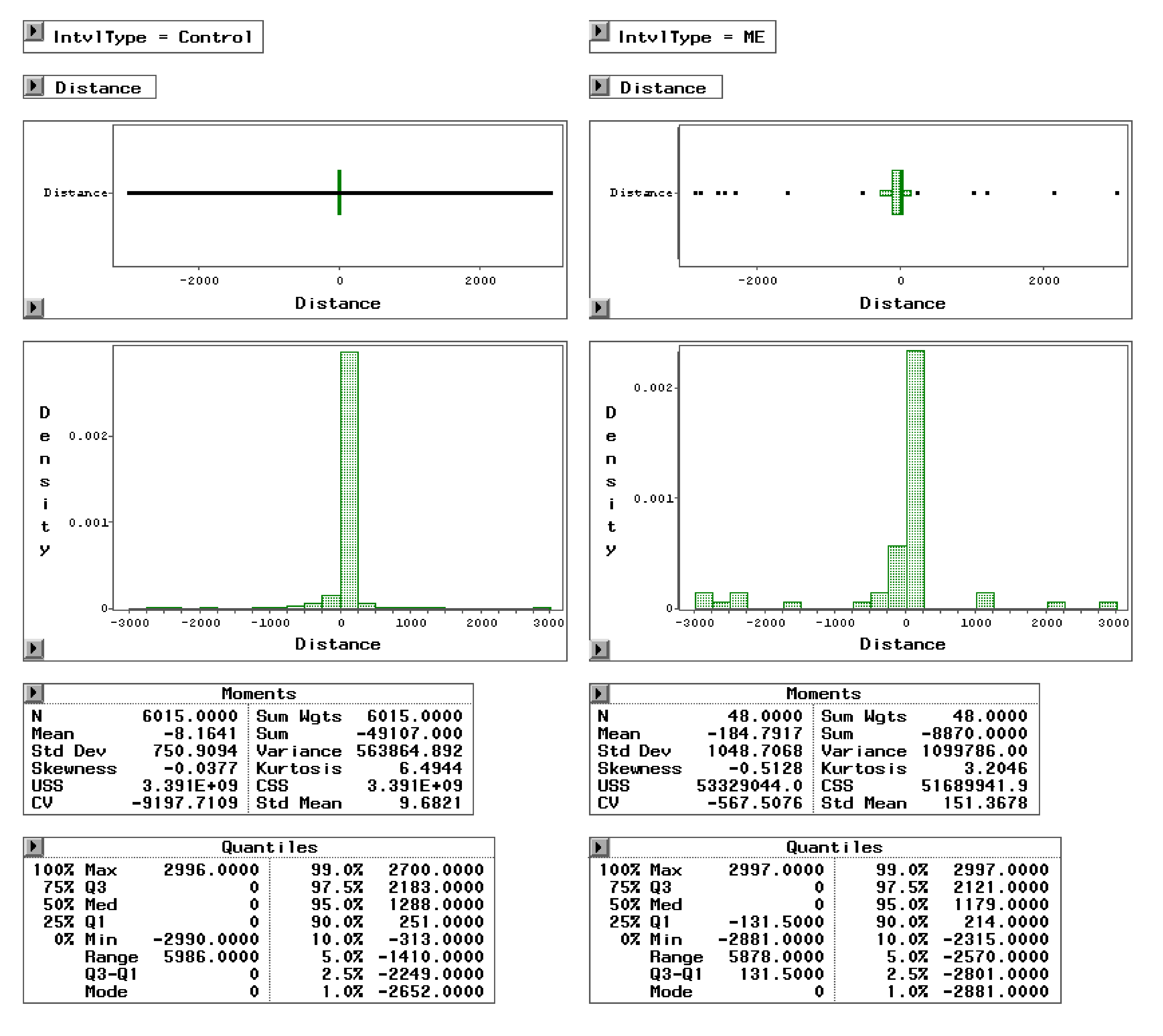

Supplement: Figure S3 — Average distance from associated CGIs is not different between control (left panel) and ME (right panel) intervals. (0.39 MB TIF) [file pgen.1001252.s003.tif]

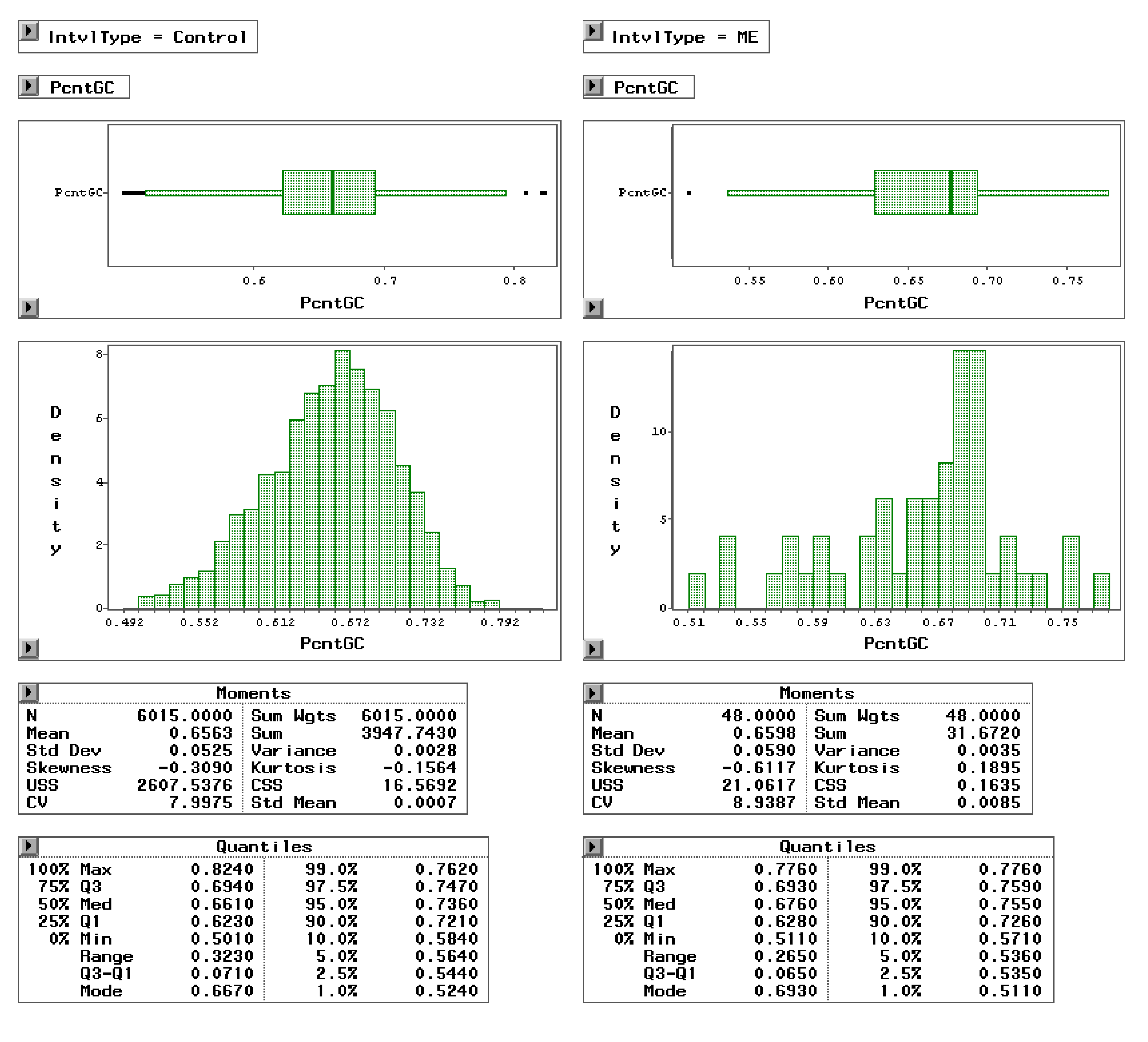

Supplement: Figure S4 — Percent GC of associated CGIs is not different between control (left panel) and ME (right panel) intervals. (0.45 MB TIF) [file pgen.1001252.s004.tif]

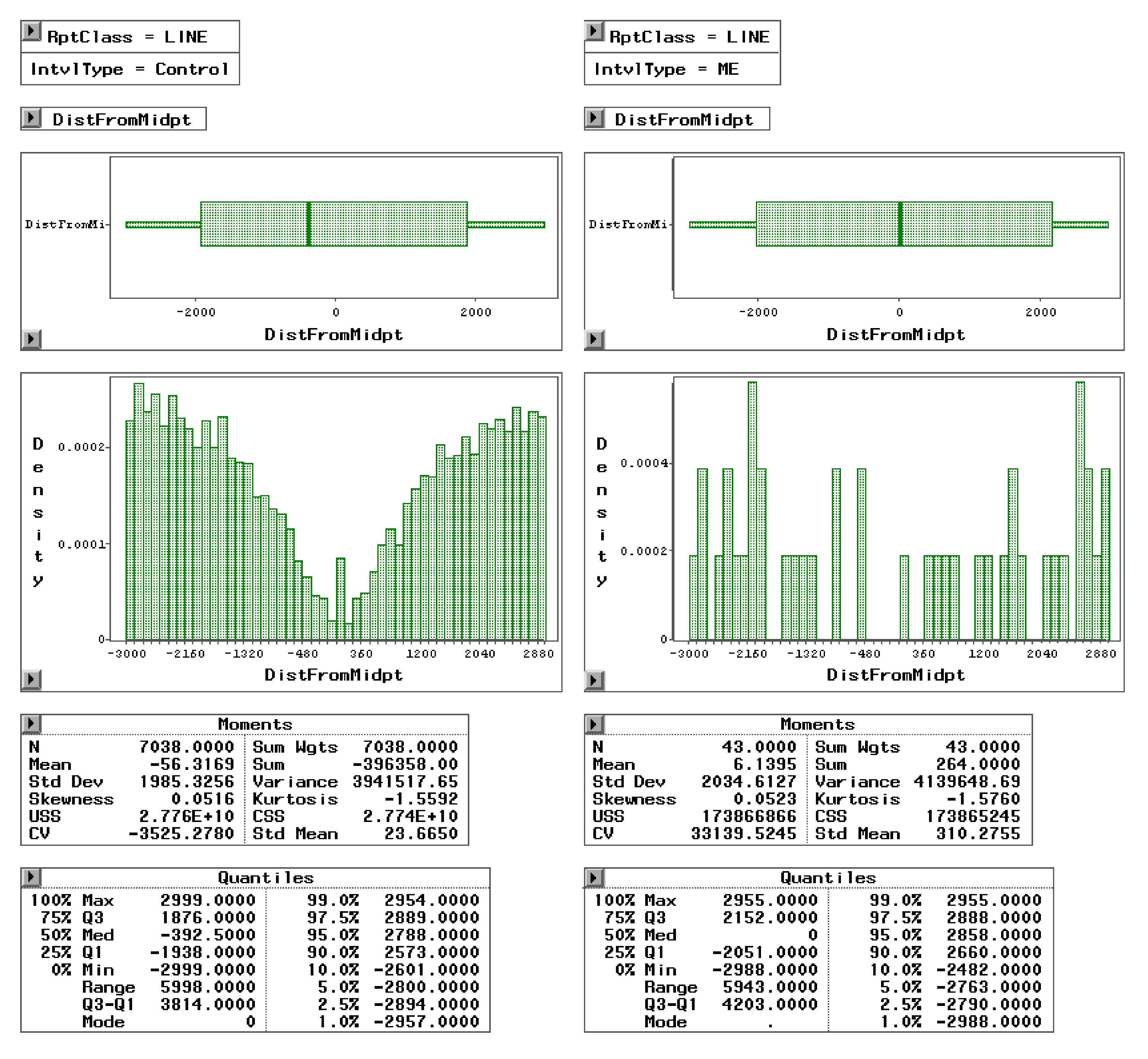

Supplement: Figure S5 — Distribution of associated LINE elements is not different between control (left panel) and ME (right panel) intervals. (0.58 MB TIF) [file pgen.1001252.s005.tif]

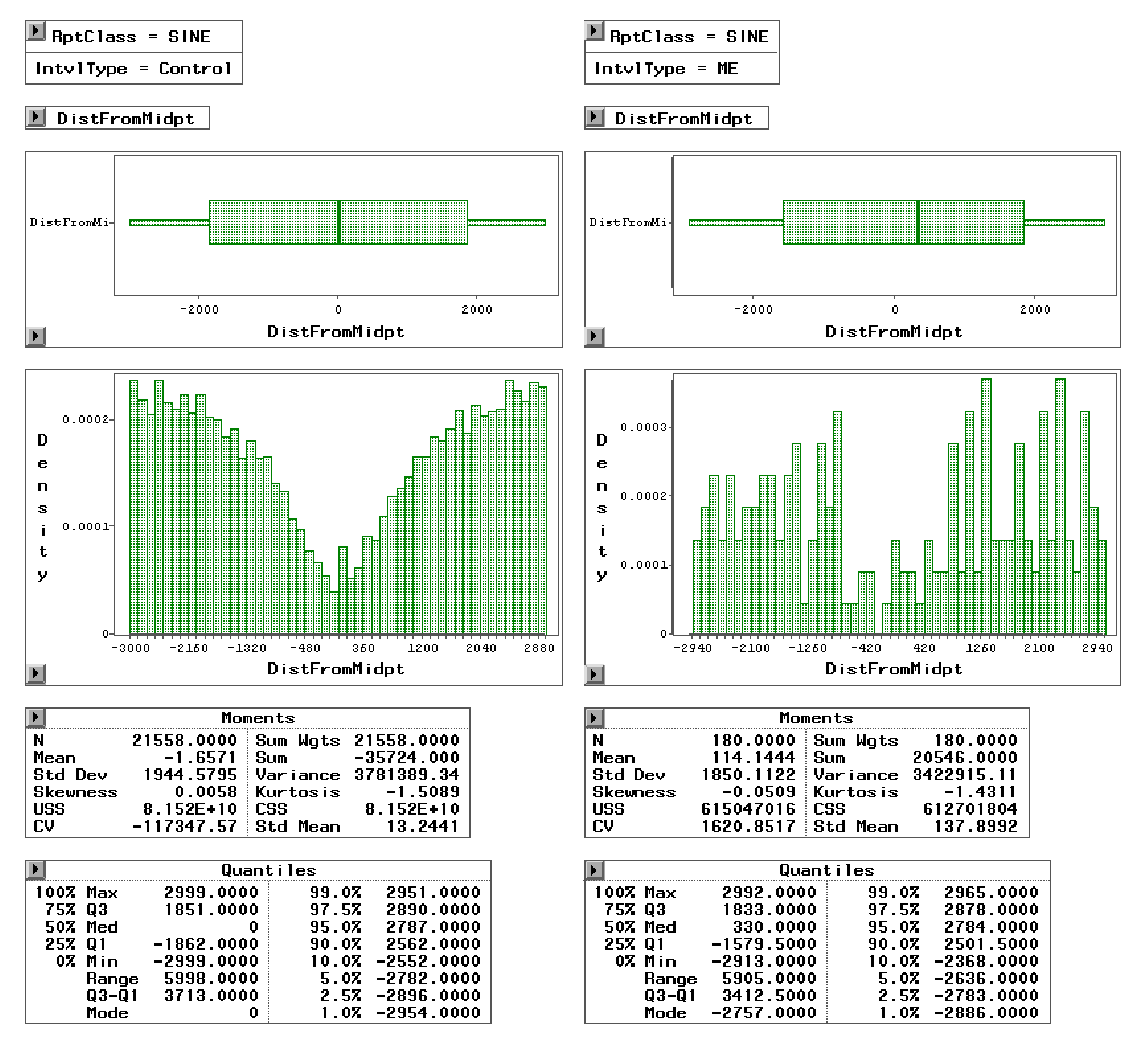

Supplement: Figure S6 — Distribution of associated SINE elements is not different between control (left panel) and ME (right panel) intervals. (0.62 MB TIF) [file pgen.1001252.s006.tif]

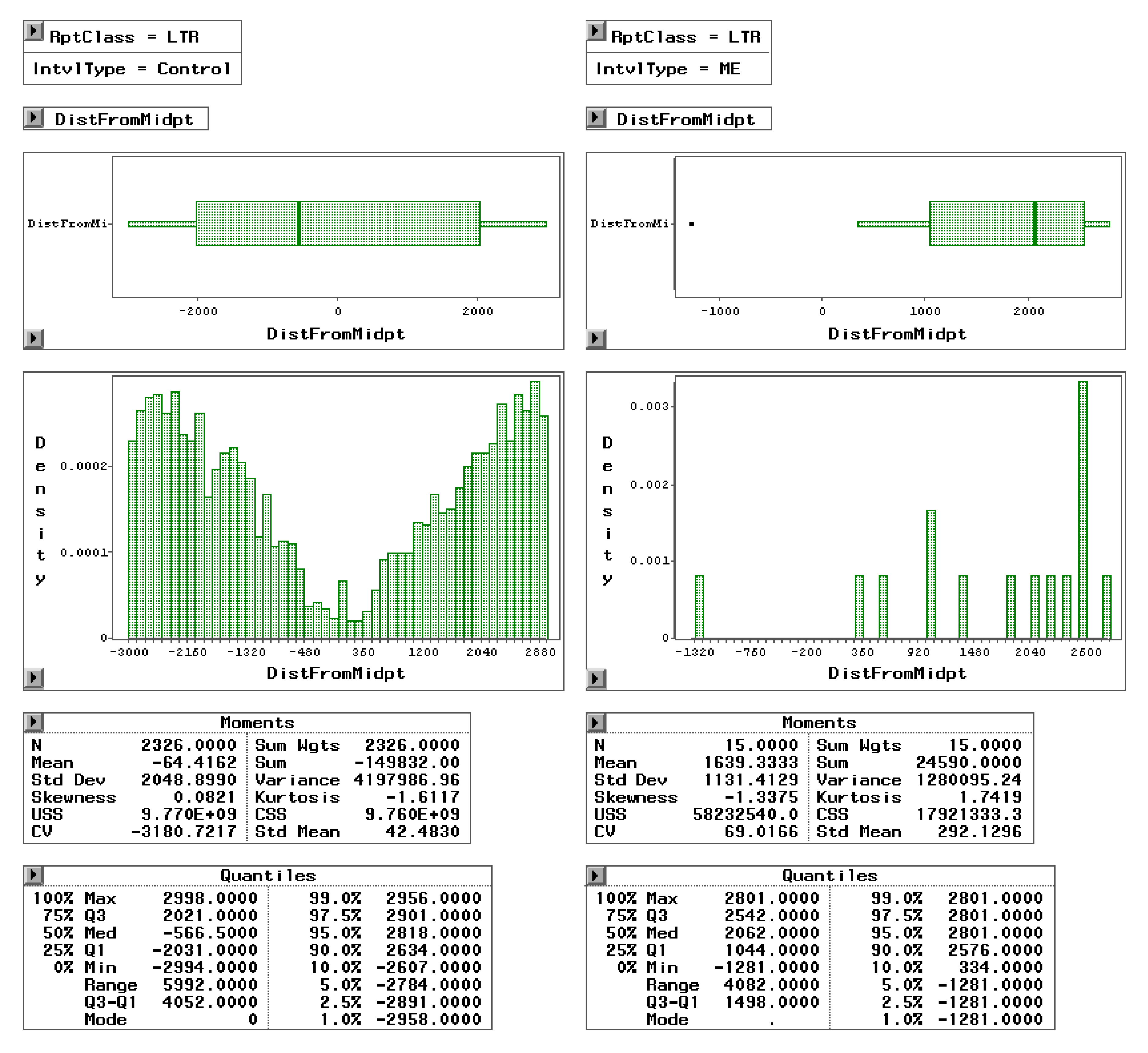

Supplement: Figure S7 — Distribution of associated LTR retrotransposons in the vicinity of control (left panel) and ME (right panel) intervals. Compared to the symmetrical distribution of those near control intervals, LTR retrotransposons occur preferentially downstream of ME intervals (P = 0.001). (0.51 MB TIF) [file pgen.1001252.s007.tif]

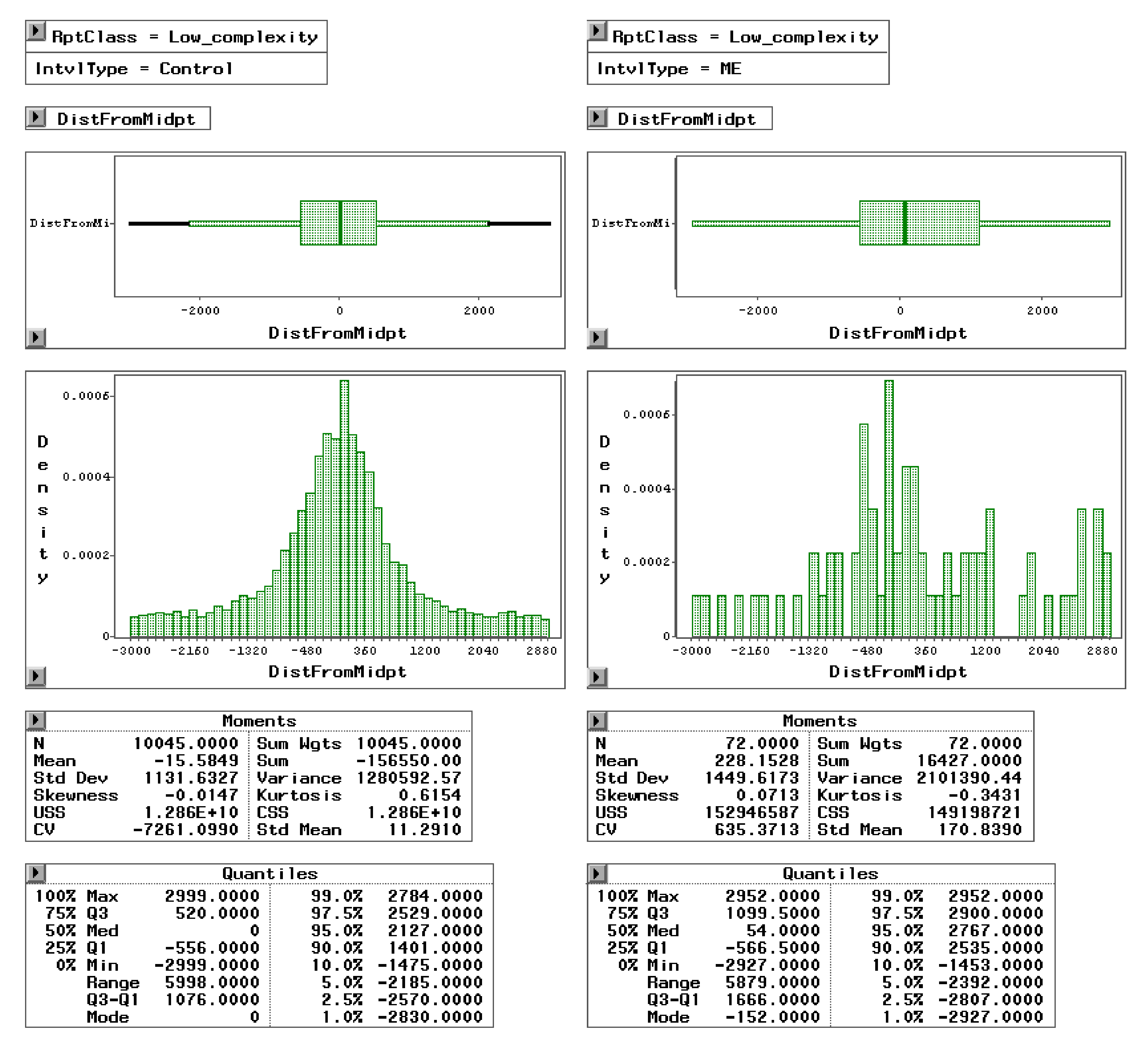

Supplement: Figure S8 — Distribution of associated low complexity repeats is not different between control (left panel) and ME (right panel) intervals. (0.49 MB TIF) [file pgen.1001252.s008.tif]

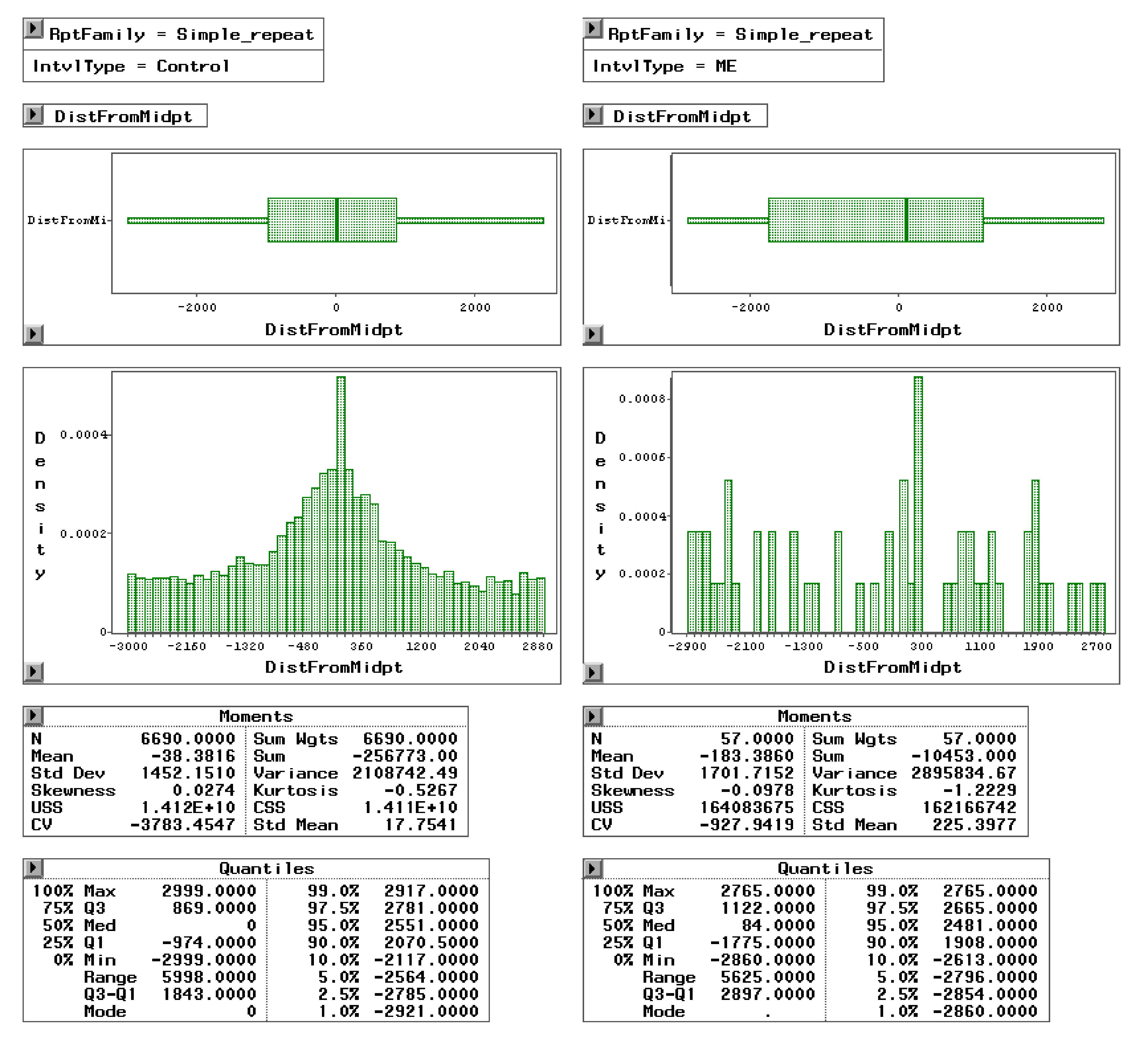

Supplement: Figure S9 — Distribution of associated simple repeats is not different between control (left panel) and ME (right panel) intervals. (0.51 MB TIF) [file pgen.1001252.s009.tif]

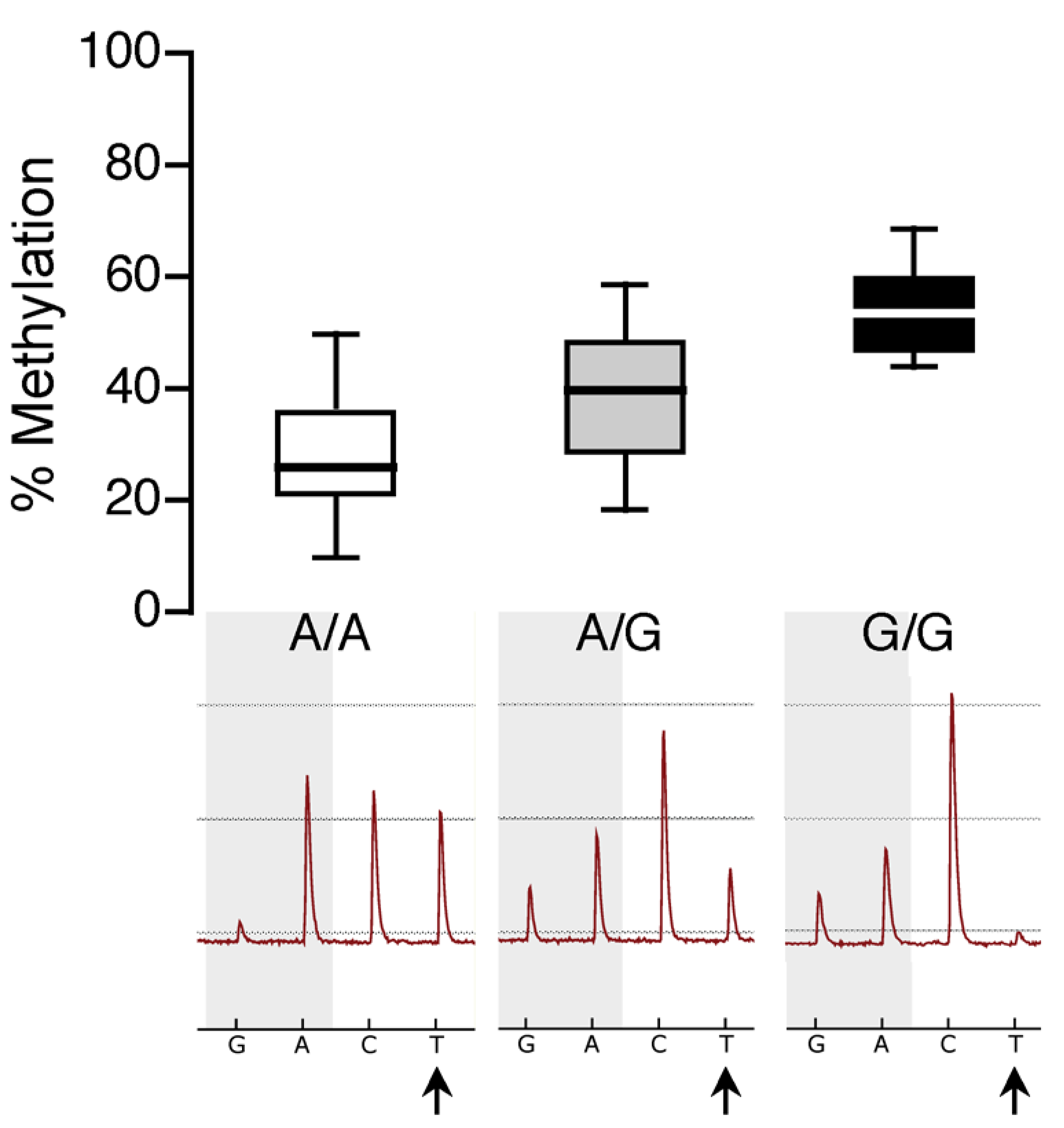

Supplement: Figure S10 — Interindividual variation in DNA methylation is predicted by genotype at ZNF696. The top panel shows average percent methylation in Gambian PBL DNA at three CpG sites measured at ZNF696 versus genotype at a neighboring A/G polymorphism (dbSNP build 130 rs28529670) (A/A, n = 25; A/G, n = 10; G/G, n = 5). The box plots indicate median (thick bar), 25th–75th percentiles (box), and 5th–95th percentiles (whiskers). The bottom panel shows representative bisulfite pyrograms for the three genotypes. A reverse sequencing primer was used; the A/G SNP is therefore detected as T/C (upward arrows). The shaded areas of the pyrograms encompass a C within a CpG site. Most interindividual variation in DNA methylation at the locus is explained by genetic variation at the A/G polymorphism. (0.52 MB TIF) [file pgen.1001252.s010.tif]

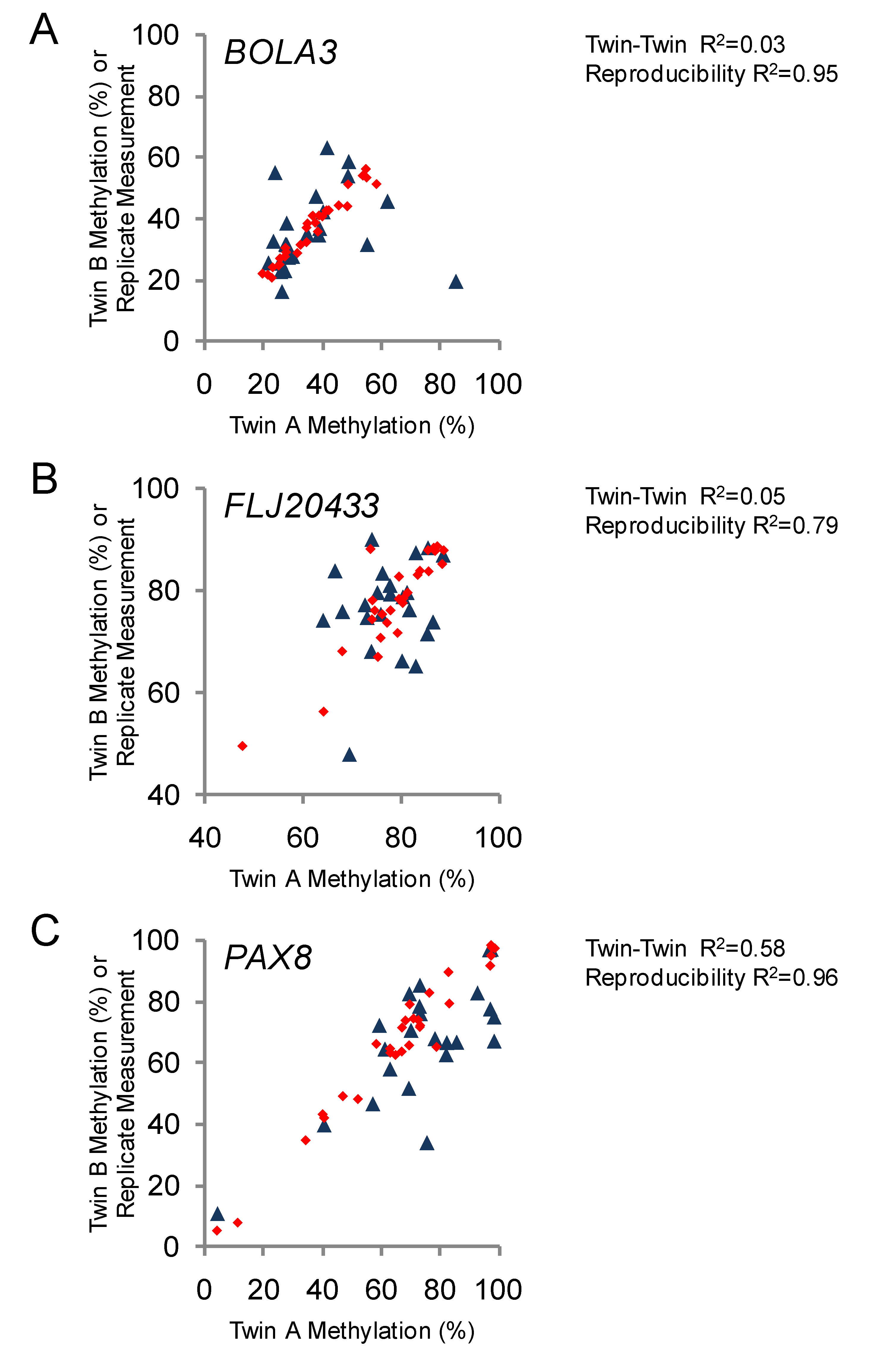

Supplement: Figure S11 — Correlation within MZ twin pairs for percent methylation at three MEs. Correlation within MZ twin pairs (blue triangles) is compared with correlation among independent replicate PCR and pyrosequencing measurements (red diamonds). Significant inter-twin correlation is found at PAX8 (C), but in every case MZ twins show biological variation that is much greater than the measurement error. (0.31 MB TIF) [file pgen.1001252.s011.tif]

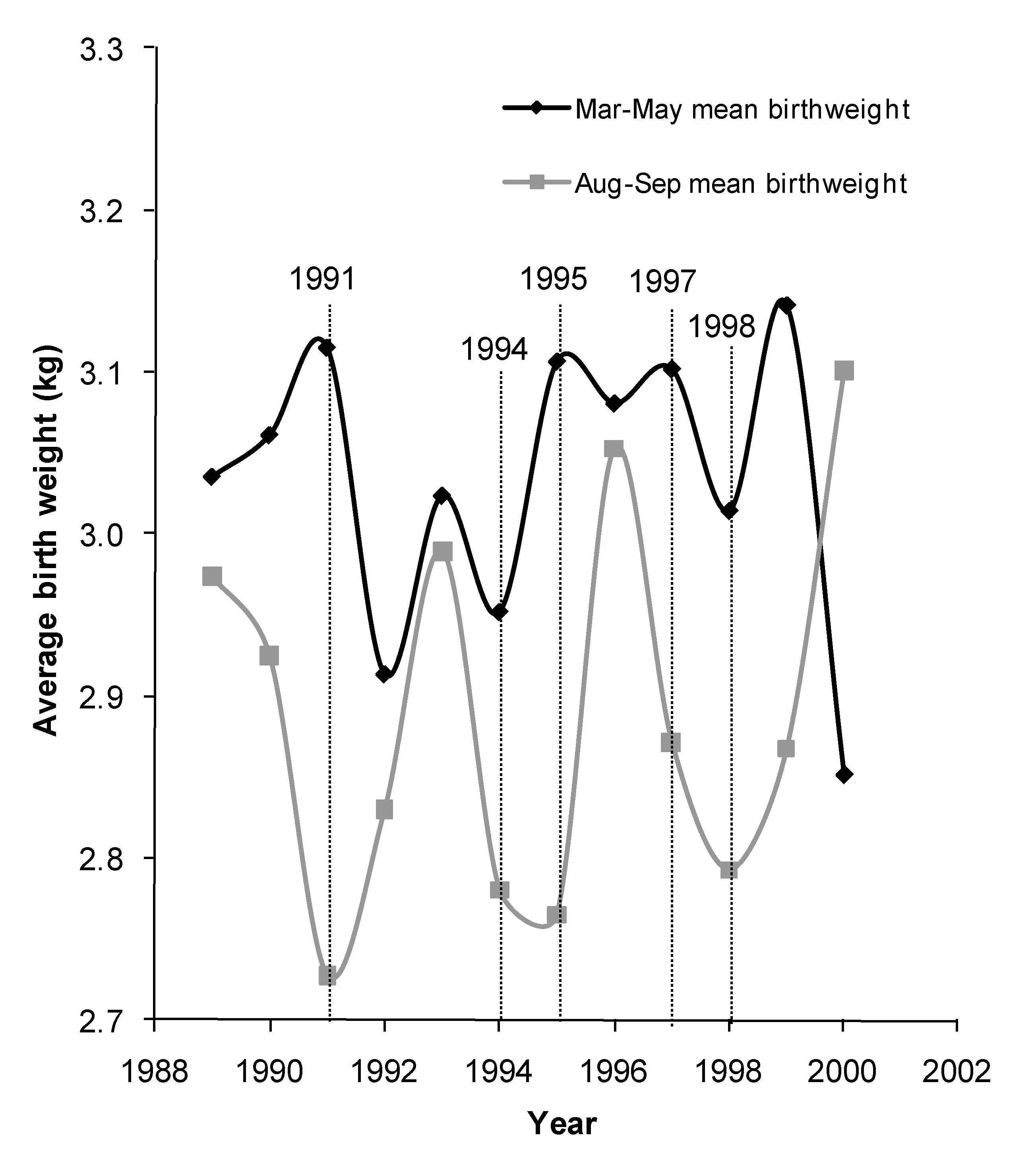

Supplement: Figure S12 — Annual variation in the effect of seasonality on birth weight in Keneba, the Gambia. Average birth weight during the peak rainy season (August-September) is compared with that during the peak dry season (March–May). Whereas some years (such as 1993) show minimal effects of season of birth, we focused on 1991, 1994, 1995, 1997, and 1998 as years with dramatic effects of seasonality. (0.20 MB TIF) [file pgen.1001252.s012.tif]

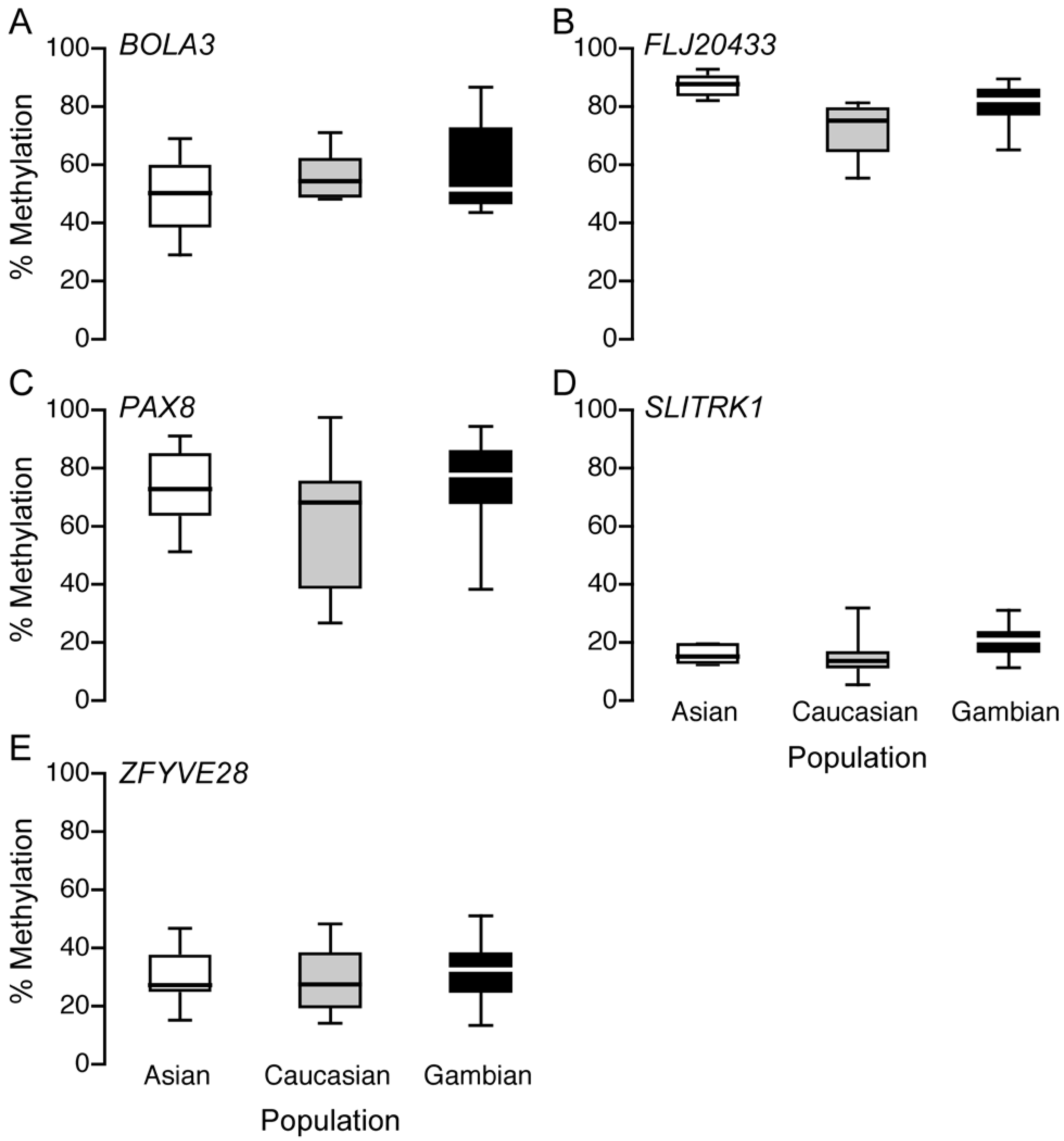

Supplement: Figure S13 — MEs exhibit similar variation in % methylation across diverse human populations. Average % methylation at BOLA3 (A), FLJ20433 (B), PAX8 (C), SLITRK1 (D), and ZFYVE28 (E) is compared across Asians (n = 8), Caucasians (n = 8), and Gambians (n = 20). The box plots indicate median (thick bar), 25th–75th percentiles (box), and 5th–95th percentiles (whiskers). Despite their genetic dissimilarity, these populations exhibit a similar range of interindividual variation in DNA methylation at each ME. (0.71 MB TIF) [file pgen.1001252.s013.tif]
