# Supplementary material for: Season of Conception in Rural Gambia Affects DNA Methylation at Putative Human Metastable Epialleles
Source: PLoS Genet. 2010 Dec 23;6(12):e1001252. doi: 10.1371/journal.pgen.1001252 (PMC3009670; doi:10.1371/journal.pgen.1001252)
Supplement: Text S1 — Detailed statistical analyses. (0.48 MB DOC) [file pgen.1001252.s023.doc]

**Detailed Statistical Analyses**

**Simultaneous analysis for ME and Control Loci, All Years Including 1997**

**Detailed Statistical Analyses, cont.**

**Simultaneous analysis for ME and Control Loci, All Years Except 1997**

**Detailed Statistical Analyses, cont.**

**Analysis for ME only, all years except 1997**

**Analysis for Control loci only, all years except 1997**
